# Supplementary material for: State-dependent protein-lipid interactions of a pentameric ligand-gated ion channel in a neuronal membrane
Source: PLoS Comput Biol. 2021 Feb 11;17(2):e1007856. doi: 10.1371/journal.pcbi.1007856 (PMC7904231; doi:10.1371/journal.pcbi.1007856)
Supplement: S1 Text — (DOCX) [file pcbi.1007856.s001.docx]

**S1 Text. Comparison of lipid binding sites to other pLGIC members.**

In current structures of GlyR, no phospholipid density has been fully resolved. However structural data for other members of pLGIC superfamily do exist and thus to further contextualize the results it is useful to make comparisons. Since these structures are more similar to the active state (open or desensitized channels), the comparison is made to data from the active state simulations. We observe 3 phospholipid interaction sites per subunit interface in the active state (see **S2 Fig**), resulting in a total of 15 phospholipid binding sites for the whole pentameric receptor.

The first phospholipid binding site is located between the M3 and M4 helices of the principal subunit with the lipid headgroup interacting with the Cys loop and coincides well with a detergent molecule site resolved in a recent GLIC crystal structure (PDB 6HZW) (see **S2 Figa**). The polar detergent headgroup forms polar interactions with the positively charged R117 and R118 side chains in the Cys loop of GLIC. Correspondingly, K143 and N144 in the human α1 GlyR make similar interactions. The hydrophobic detergent tail in the GLIC structure interacts with the membrane facing surface of the M3 and M4 helices and coincides with the lipid position in our simulations. A second interaction site occurs between the M1 and M4 helices of the complementary subunit with the head group interacting with the pre-M1 region. This binding site is similar to that found in GLIC (PDB 6HZW) as well as in a recent GABA_A_ receptor cryo-EM structure (PDB 6I53), although the structurally resolved binding modes differ slightly among each other and with regard to the density observed in our simulations. In GLIC (see **S2 Figb**), a phosphocholine molecule is bound between the M1 and the M4 helix. Note that the M4 helix in the GLIC structure is kinked slightly more away from the M1/M3 interface than in the human α1 GlyR, allowing the phosphocholine molecule to insert itself deeper in between the M4-M1/M3 helix interface, while the phospholipid density in our simulations does not penetrate this interface, but rather, is located at the membrane facing surface of the M1 and M4 helices. The looser contact of the M4 helix with the M1/M3 helix interface in GLIC can be explained in terms of less aromatic residues that hold the M4 helix close to the M1/M3 helices in the human α1 GlyR. Moreover, the phosphocholine insertion at this site in GLIC is further supported by an electrostatic interaction of the phosphate headgroup with R118 on the M2-M3 linker, whereas the human α1 GlyR has an asparagine at this position which cannot engage in such an interaction due to its much shorter side chain and lack of charge.

In the human GABA_A_R cryo-EM structure (PDB 6I53), two slightly different phospholipid binding modes are observed in this region. **S2 Figc** shows a phospholipid bound to the α1 subunit that is positioned slightly more towards the subunit interface, mainly interacting with the M1 helix via its lipid tails with its phosphate headgroup stabilized via electrostatic interactions with K222 in the pre-M1 region. We show an alternative mode of a phospholipid bound to the GABA_A_R β3 subunit (**S2 Figd**) whose lipid tails are located on the membrane facing surface of the M1 and M4 helices and its phosphate headgroup can engage in electrostatic interactions with K215 in the pre-M1 region. The phospholipid modes in the human GABA_A_R cryo-EM structure are similar to the phospholipid densities observed in our simulations of the active human α1 GlyR in the sense that the lipid tail positions are located on the membrane facing surfaces of the M1 and M4 helices, rather than penetrating the M4-M1/M3 interface as seen in the GLIC structure (**S2 Figb**).

The third interaction site is at the subunit interface between the (+)M3 and (-)M1 helices with the lipid headgroup interacting with the (+)M2-M3 linker, in manner not previously observed in any pLGIC structure and may be particular to the GlyR.
