# Supplementary material for: State-dependent protein-lipid interactions of a pentameric ligand-gated ion channel in a neuronal membrane
Source: PLoS Comput Biol. 2021 Feb 11;17(2):e1007856. doi: 10.1371/journal.pcbi.1007856 (PMC7904231; doi:10.1371/journal.pcbi.1007856)
Supplement: S1 Fig — Exemplary data shown for 4 DOPS molecules from repeat 1 of the inactive state simulations. Each scatter plot illustrates the movement of a single DOPS molecule over 20 μs of simulation (last 20 μs out of 40 μs total simulation time). Each circle in the scatter plots represents the position of a single DOPS molecule at every 10 ns of simulation time (a transparent shading is used for the circles, so as to highlight preferred lipid locations resulting in darker colours from overlapping circles). Clearly, in a single simulation repeat a single DOPS molecule leaves the receptor surface multiple times and samples large areas of both receptor surface as well as in the membrane (See also Fig 2 of the main manuscript). (PDF) [file pcbi.1007856.s002.pdf]

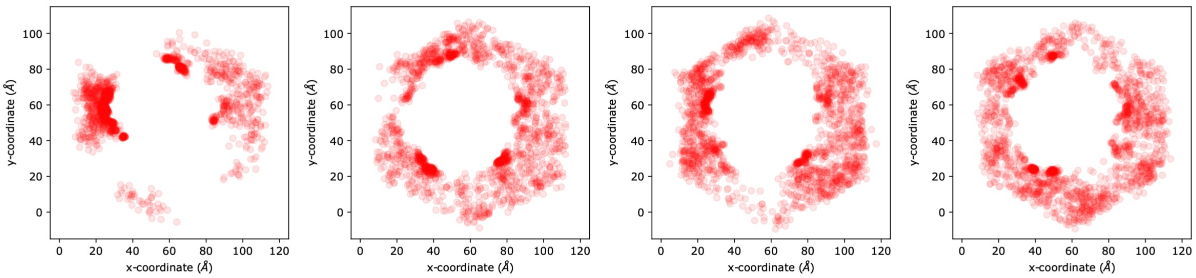

S1 Fig. Trajectories of single DOPS molecules in the membrane plane for the inactive state

Exemplary data shown for 4 DOPS molecules from repeat 1 of the inactive state simulations. Each scatter plot illustrates the movement of a single DOPS molecule over 20 μs of simulation (last 20 μs out of 40 μs total simulation time). Each circle in the scatter plots represents the position of a single DOPS molecule at every 10 ns of simulation time (a transparent shading is used for the circles, so as to highlight preferred lipid locations resulting in darker colours from overlapping circles). Clearly, in a single simulation repeat a single DOPS molecule leaves the receptor surface multiple times and samples large areas of both receptor surface as well as in the membrane. (See also Figure 2 of the main manuscript).
