# Supplementary material for: State-dependent protein-lipid interactions of a pentameric ligand-gated ion channel in a neuronal membrane
Source: PLoS Comput Biol. 2021 Feb 11;17(2):e1007856. doi: 10.1371/journal.pcbi.1007856 (PMC7904231; doi:10.1371/journal.pcbi.1007856)
Supplement: S2 Fig — The colour scales were chosen to highlight the state-dependent differences per leaflet and lipid type. Darker colours represent interaction hotspots. The initial positions of lipid molecules in each simulation repeat were assigned randomly in order to generate different starting conditions and thus ensure overall a better sampling of lipid mixing. Similar interaction hot spots emerge in the individual simulation repeats, although with different intensity due to the limited sampling time of each individual simulation repeat. Averaging over all 10 simulation repeats, however, yields a rather converged density map of the lipid distribution. (See also Fig 2 of the main manuscript). (PDF) [file pcbi.1007856.s003.pdf]

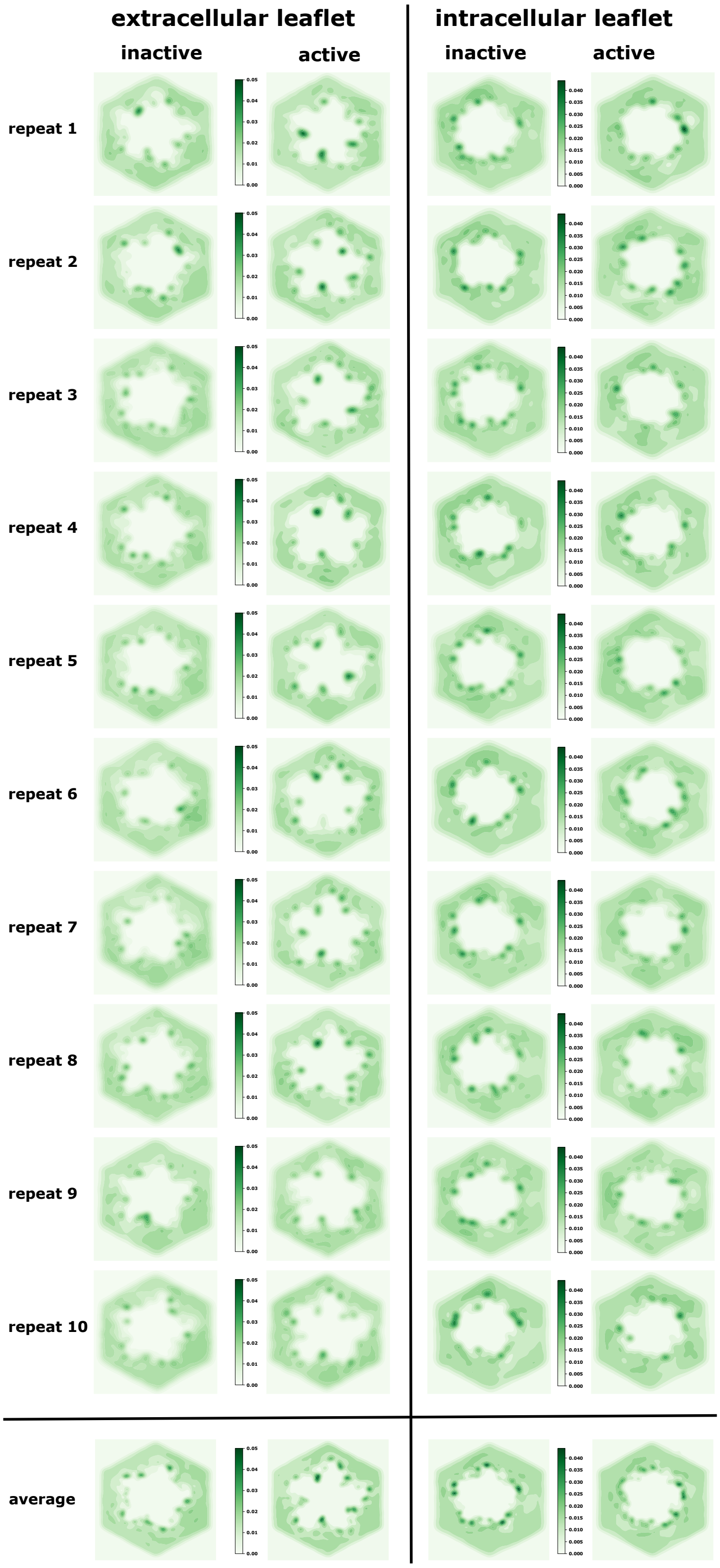

S2 Fig. Probability densities of cholesterol for each individual simulation repeat around the receptor in the extracellular and intracellular leaflet for the inactive and active state

The colour scales were chosen to highlight the state-dependent differences per leaflet and lipid type. Darker colours represent interaction hotspots. The initial positions of lipid molecules in each simulation repeat were assigned randomly in order to generate different starting conditions and thus ensure overall a better sampling of lipid mixing. Similar interaction hot spots emerge in the individual simulation repeats, although with different intensity due to the limited sampling time of each individual simulation repeat. Averaging over all 10 simulation repeats, however, yields a rather converged density map of the lipid distribution. (See also Figure 2 of the main manuscript).
