# Supplementary material for: State-dependent protein-lipid interactions of a pentameric ligand-gated ion channel in a neuronal membrane
Source: PLoS Comput Biol. 2021 Feb 11;17(2):e1007856. doi: 10.1371/journal.pcbi.1007856 (PMC7904231; doi:10.1371/journal.pcbi.1007856)
Supplement: S3 Fig — Averaged over last 20 μs (out of 40μs) of 10 repeats per conformational state and over the 5 subunits. A cut-off of 6 Å is used, which corresponds to the first lipid shell. (PDF) [file pcbi.1007856.s004.pdf]

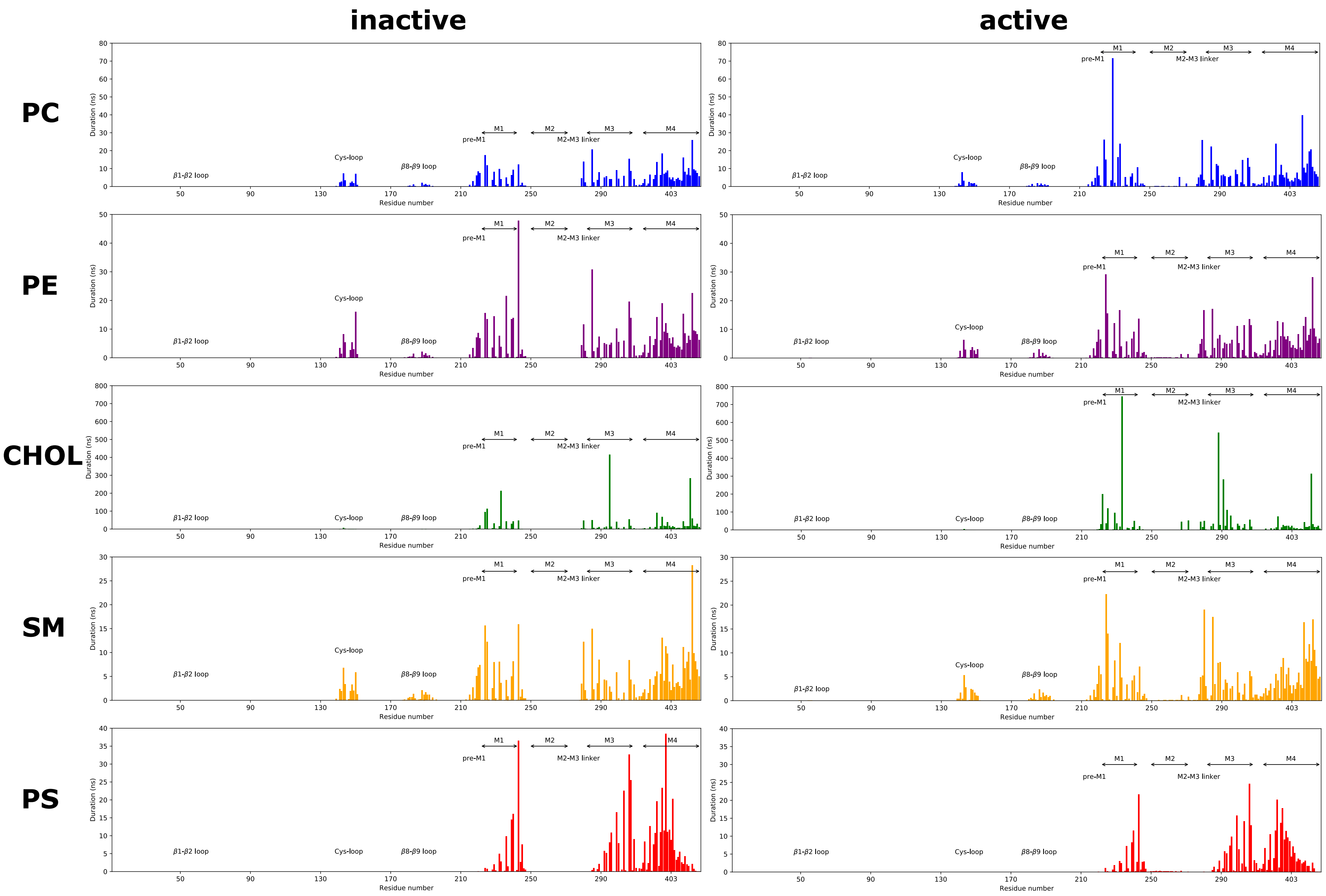

S3 Fig. Mean duration of protein-lipid contact per residue for inactive and active state

Averaged over last 20 μs (out of 40μs) of 10 repeats per conformational state and over the 5 subunits. A cut-off of 6 Å is used, which corresponds to the first lipid shell.
