# Supplementary material for: State-dependent protein-lipid interactions of a pentameric ligand-gated ion channel in a neuronal membrane
Source: PLoS Comput Biol. 2021 Feb 11;17(2):e1007856. doi: 10.1371/journal.pcbi.1007856 (PMC7904231; doi:10.1371/journal.pcbi.1007856)
Supplement: S4 Fig — Overlays of the human α1 GlyR model (darker colours) and phospholipid density (transparent, phosphate headgroup density in red, choline/ammonium headgroup density in blue, tail density in cyan) from simulations in the active state with experimentally resolved structures of the pLGIC superfamily (lighter colours) with phospholipid or detergent bound. Three binding sites are observed in simulations (labelled 1, 2 and 3). Lipid binding has been observed in the corresponding regions of sites 1 and 2 for other members of the pLGIC family, but site 3 may be specific to the GlyR. a Overlay with GLIC crystal structure (PDB 6HZW) which has a detergent molecule bound that interacts with its polar headgroup with the Cys loop, while its hydrophobic tail mainly interacts with the M3 helix. b Overlay with GLIC crystal structure (PDB 6HZW) which has a phosphocholine lipid bound that interacts with its polar headgroup with the pre-M1 region as well as the Cys loop, while its hydrophobic tails bridge the interface between the M4 and M1/M3 helices. c Overlay with the α1 subunit of the human GABAA receptor cryo-EM structure (PDB 6I53), which has a phospholipid bound that interacts with its polar headgroup with the pre-M1 region, while its hydrophobic tails interact mainly with the M1 helix. d Overlay with the β3 subunit of the human GABAA receptor cryo-EM structure (PDB 6I53), which has a phospholipid bound that interacts with its polar headgroup with the pre-M1 region, while its hydrophobic tails interact with both the M1 and M4 helices. (PDF) [file pcbi.1007856.s005.pdf]

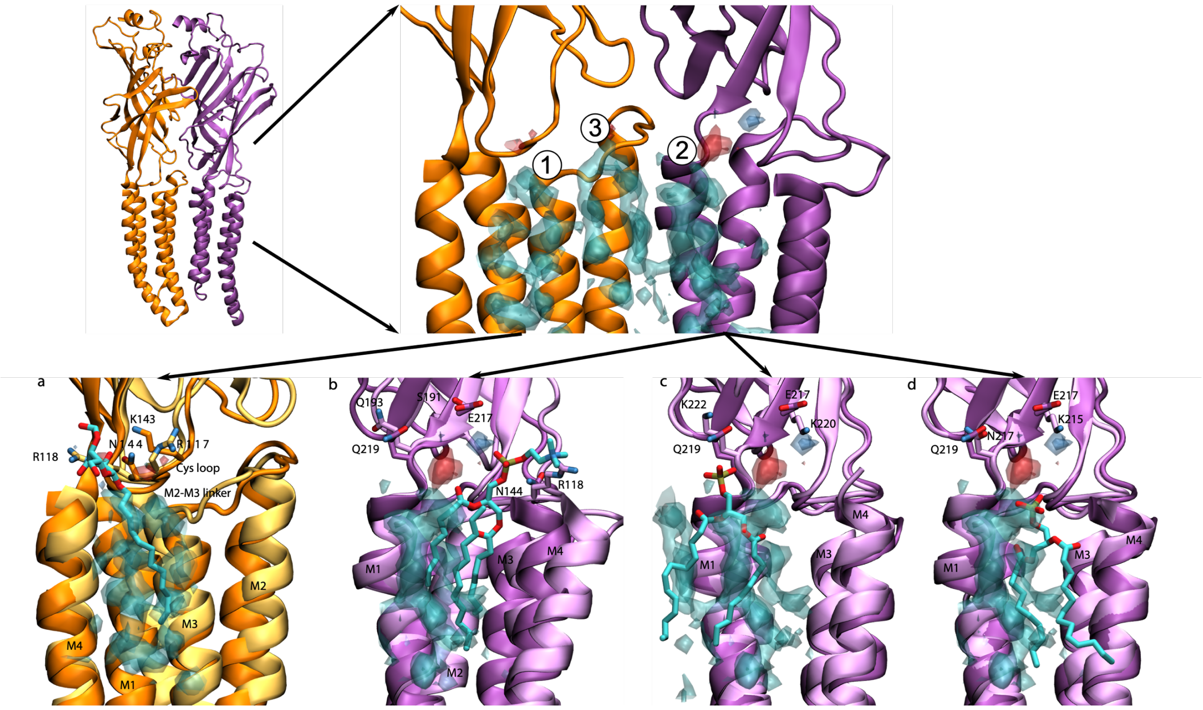

S4 Fig. Overlay of phospholipid densities at the ECD-TMD interface with experimental structures

Overlays of the human α1 GlyR model (darker colours) and phospholipid density (transparent, phosphate headgroup density in red, choline/ammonium headgroup density in blue, tail density in cyan) from simulations in the active state with experimentally resolved structures of the pLGIC superfamily (lighter colours) with phospholipid or detergent bound. Three binding sites are observed in simulations (labelled 1, 2 and 3). Lipid binding has been observed in the corresponding regions of sites 1 and 2 for other members of the pLGIC family, but site 3 may be specific to the GlyRs **a** Overlay with GLIC crystal structure (PDB 6HZW) which has a detergent molecule bound that interacts with its polar headgroup with the Cys loop, while its hydrophobic tail mainly interacts with the M3 helix. **b** Overlay with GLIC crystal structure (PDB 6HZW) which has a phosphocholine lipid bound that interacts with its polar headgroup with the pre-M1 region as well as the Cys loop, while its hydrophobic tails bridge the interface between the M4 and M1/M3 helices. **c** Overlay with the α1 subunit of the human $GABA_A$ receptor cryo-EM structure (PDB 6I53), which has a phospholipid bound that interacts with its polar headgroup with the pre-M1 region, while its hydrophobic tails interact mainly with the M1 helix. **d** Overlay with the β3 subunit of the human $GABA_A$ receptor cryo-EM structure (PDB 6I53), which has a phospholipid bound that interacts with its polar headgroup with the pre-M1 region, while its hydrophobic tails interact with both the M1 and M4 helices.
