# Supplementary material for: State-dependent protein-lipid interactions of a pentameric ligand-gated ion channel in a neuronal membrane
Source: PLoS Comput Biol. 2021 Feb 11;17(2):e1007856. doi: 10.1371/journal.pcbi.1007856 (PMC7904231; doi:10.1371/journal.pcbi.1007856)
Supplement: S5 Fig — Darker colours: moving average, lighter colours: original, unsmoothed values. Both states exhibit a similar, low level of flexibility and remain close (around 2–3 Å) to the starting model, supporting the high quality of the homology models. (PDF) [file pcbi.1007856.s006.pdf]

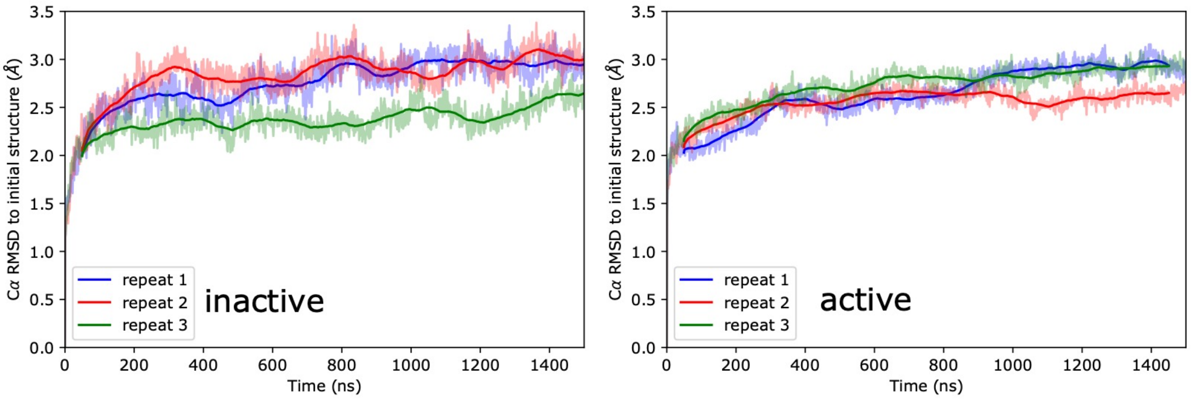

S5 Fig. Cα-RMSD of inactive and active state homology models in fully atomistic simulations

Darker colours: moving average, lighter colours: original, unsmoothed values. Both states exhibit a similar, low level of flexibility and remain close (around 2-3 Å) to the starting model, supporting the high quality of the homology models.
