# Supplementary material for: State-dependent protein-lipid interactions of a pentameric ligand-gated ion channel in a neuronal membrane
Source: PLoS Comput Biol. 2021 Feb 11;17(2):e1007856. doi: 10.1371/journal.pcbi.1007856 (PMC7904231; doi:10.1371/journal.pcbi.1007856)
Supplement: S6 Fig — The data for the position-restrained approach (top 4 panels) is also presented in Fig 2 in the main text. The data for the elastic network approach (bottom 4 panels) is based on 5 simulation repeats of 2 μs length for each the active and inactive simulation systems. Similar lipid locations for the position-restrained and elastic network approach can be observed. This substantiates the assumption that both approaches lead to similar lipid distributions. (PDF) [file pcbi.1007856.s007.pdf]

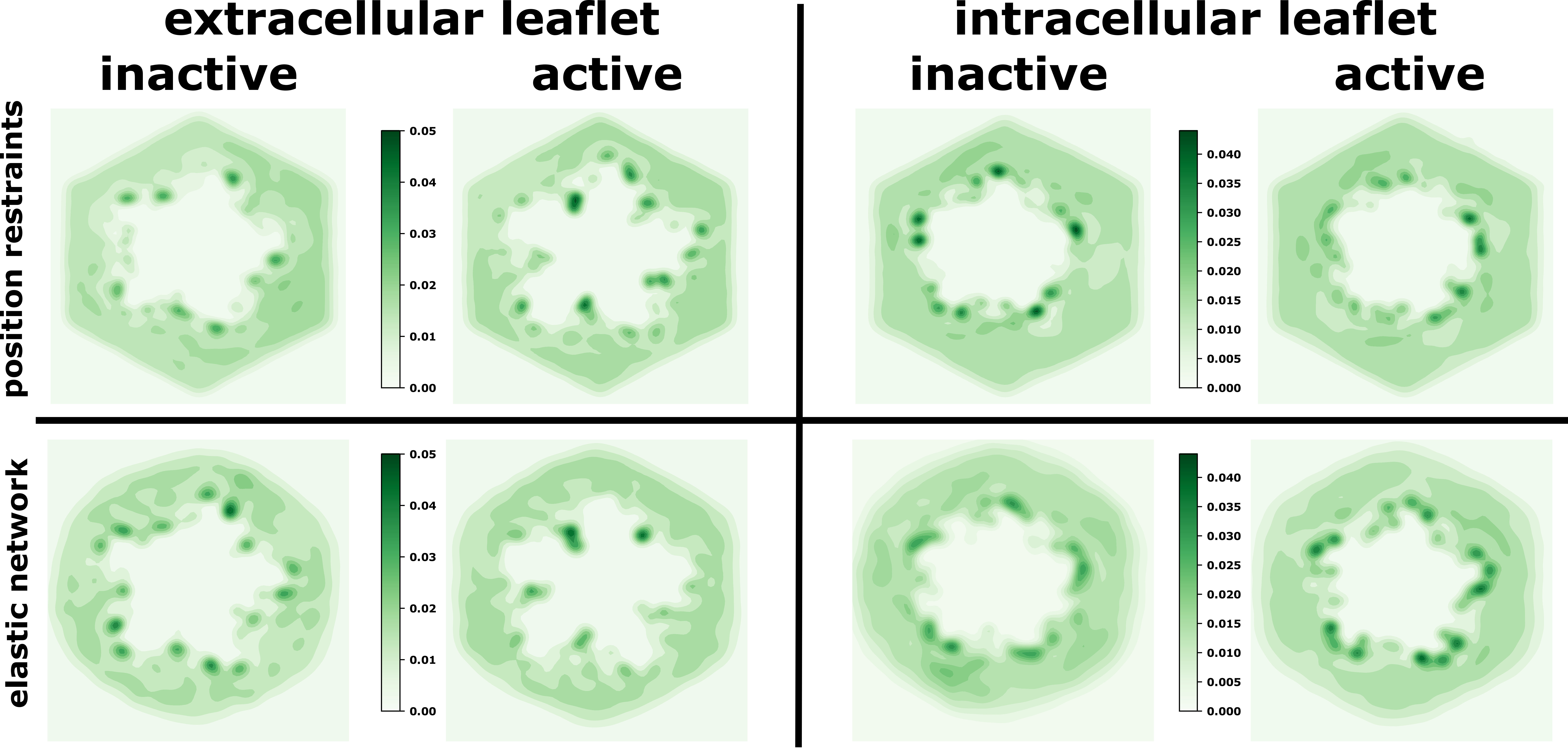

extracellular leaflet
intracellular leaflet
inactive
active
inactive
active
position restraints
elastic network
0.00
0.01
0.02
0.03
0.04
0.05
0.000
0.005
0.010
0.015
0.020
0.025
0.030
0.035
0.040
